# Editorial

**Published:** 2013-04-26

**Authors:** Nikhil Marwah

## Abstract

In recent years, there has been increasing pressure on postdoctoral students and teachers to write or coauthor scientific publications which govern their postdoctoral opportunities, grants and even employment.

## The Author Files: Who should be the First Author...

In recent years, there has been increasing pressure on postdoctoral students and teachers to write or coauthor scientific publications which govern their postdoctoral opportunities, grants and even employment. This has started a race, a race to be first. Social forums across the globe are ripe with conversations regarding the pedigree that has to be followed while sequencing the authors. While some believe the guide should be the first author as there are others who believe that the person who has done the research should be the first author. Some of the historical approaches in sequencing of authors are as follows:

- *Sequence determines credit approach:* The sequence of authors should reflect the declining importance of their contribution.
- *Equal contribution norm:* Authors use alphabetical sequence to acknowledge similar contributions.
- *Percent contribution indicated approach:* Each author's contribution quantified as percentage in the article.
- *Alphabetic authorship approach:* Used to denote a large teamwork like in astronomy, oceanographical surveys and long-term medical surveys.

One of the most distracting issues in scientific publishing is deciding who gets the credit as a first author and what will be the order of other coauthors. The second vital issue faced by most authors is honorary authorship (named authors who do not fit into authorship criteria). The main hypothesis for this usually is unequal power relations where junior researchers may feel pressured to accept being second author inspite of actually being the first, so as to oblige their superiors who have substantial power over their future career; for repaying favors; encouraging collaboration and maintaining good working relationships. To avoid such bias in the authorship, the criterion for authorship is outlined by International Committee of Medical Journal Editors (ICMJE) in Uniform Requirements for Manuscripts Submitted to Biomedical Journals (Updated October 2004 Available from: http://www.icmje.org).

- Authorship credit should be based on substantial contributions to conception and design, acquisition of data or analysis and interpretation of data; drafting the article or revising it critically for important intellectual content and final approval of the version to be published.
- Authorship should be reserved for those and only those, who have made significant intellectual contribution to the research. Acquisition of funding, collection of data or general supervision of the research group alone does not constitute authorship.
- The sequence determines credit approach is the most justified norm for authorship.
- The person who has made the major contribution to the paper and/or taken the lead in writing is entitled to be the first author. Coauthors are contributors to the development of the project and usually provide their academic contributions as designated by the first author. All contributors who do not meet the criteria for authorship should be listed in an acknowledgments section.
- Postdoctoral students should be the first authors of journal articles based on their thesis or dissertation manuscripts as per the scientific guidelines.
- Each manuscript should contain a clear description of each author's contribution in the article.

I think it is time to lay the ghosts to rest and look at the bare facts and start practicing in accordance with the norms.

                                           …Give The Credit Where It Is Due...


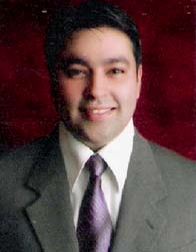
**Nikhil Marwah**
Editor-in-chief


